# An avian influenza A (H7N9) virus vaccine candidate based on the fusion protein of hemagglutinin globular head and *Salmonella typhimurium* flagellin

**DOI:** 10.1186/s12896-015-0195-z

**Published:** 2015-08-19

**Authors:** Li Song, Dan Xiong, Xilong Kang, Yun Yang, Jing Wang, Yaxin Guo, Hui Xu, Sujuan Chen, Daxin Peng, Zhiming Pan, Xinan Jiao

**Affiliations:** Jiangsu Co-innovation Center for Prevention and Control of Important Animal Infectious Diseases and Zoonoses, Yangzhou, Jiangsu 225009 China; Jiangsu Key Laboratory of Zoonosis, Yangzhou University, 48 East Wenhui Road, Yangzhou, Jiangsu 225009 China

## Abstract

**Background:**

A novel influenza virus, subtype H7N9, circulated through China in 2013–2014. Its higher rates of human infection in a wide range of locations within China and the associated increased likelihood of human-to-human transmission have caused global concern. Recombinant subunit vaccines provide safe and targeted protection against viral infections. However, the protective efficacy of recombinant subunit vaccines tends to be less potent than vaccines made from whole viruses. Studies have shown that bacterial flagellin has strong adjuvant activity and induces protective immune responses.

**Results:**

In this study, we used overlap-PCR to generate an H7N9 influenza recombinant subunit vaccine that fused the globular head domain (HA1-2, aa 62–284) of the protective hemagglutinin (HA) antigen with the potent TLR5 ligand, *Salmonella typhimurium* flagellin (fliC). The resulting fusion protein, HA1-2-fliC, was efficiently expressed in an *Escherichia coli* prokaryotic expression system, and Western blotting and TLR5-stimulating activity analysis confirmed that the HA1-2-fliC moiety could be faithfully refolded to take on the native HA and fliC conformations. In a C3H/HeJ mouse model of intraperitoneal vaccination, the fusion protein elicited significant and robust HA1-2-specific serum IgG titers, maintaining high levels for at least 3 months in the vaccinated animals, and induced similar levels of HA1-2-specific IgG1 and IgG2a that were detectable 12 days after the third immunization. HA1-2-fliC was also found to be capable of triggering the production of neutralizing antibodies, as assessed by measuring hemagglutination inhibition titers.

**Conclusions:**

We conclude that immunization with HA1-2-fliC induces potent HA1-2-specific responses, offering significant promise for the development of a successful recombinant subunit vaccine for avian influenza A (H7N9).

## Background

Avian-origin influenza A (H7N9) virus emerged as a human pathogen in China in spring 2013 and, as of October 2014, it caused 453 human cases and 175 deaths [1]. At this rate, it will soon match or surpass the burden of avian influenza A (H5N1) (676 cases, as of December 2014) [2]. Cases of H7N9 are currently accumulating at a pace that is five times faster than avian influenza A (H5N1). The epidemiology of this outbreak has implied that live bird markets are the source of human infections. Although shutting down live poultry markets resulted in an immediate reduction in cases [3], the outbreak of human infection with influenza H7N9 virus has re-emphasized the importance of making faster and more effective influenza vaccines than those that are currently available.

At present, live-attenuated, inactivated whole virus or split vaccines produced in embryonated hens’ eggs are used to control influenza. However, production of these types of H7N9 influenza vaccines often has several hurdles [4]. Some laboratories have studied the live-attenuated H7N9 virus vaccine candidate [5] and other H7 subtype virus vaccines [6, 7] for their ability to protect from H7N9 virus infection.

Subunit vaccines, in contrast to live-attenuated or inactivated whole virus vaccines, include only specific antigens that stimulate immune responses. Recombinant subunit vaccine technology is a promising approach to develop safe vaccines capable of inducing specific immune responses [8]. However, compared with live-attenuated or inactivated whole virus vaccines, subunit vaccines have low immune-stimulating capacity. It is now well established that linkage of Toll-like receptor (TLR) ligands and vaccine antigens enhances the immunopotency of the linked antigen [9].

Flagellin, a TLR5 ligand, induces downstream signaling in a MyD88-dependent manner [10]. Studies from several groups have established that recognition of flagellin by the innate immune system leads to cytokine production and dendritic cell (DC) activation [11–13]. The adjuvant effect of flagellin has been demonstrated in a variety of pathogen models, including influenza [14, 15], *Yersinia pestis* [16], and *Pseudomonas aeruginosa* [17]. These protective responses characteristically exhibit remarkably high titers of antigen-specific IgG.

Hemagglutinin (HA), the surface glycoprotein of influenza virus, has been the key protective antigen in seasonal influenza vaccine studies for 40 years [14]. The overall predicted HA protein structure of A/Hangzhou/1/2013 (H7N9), closely resembles other reported HA structures [18]. The HA globular head domain contains the cell surface receptor binding site and the majority of the neutralizing antibody epitopes [19, 20]. Studies have shown that HA1-2 (residues 62–284) on the HA globular head domain encompasses the neutralizing epitopes of the globular head and also contains the structural elements necessary for efficient folding to correctly display these epitopes after recombinant protein expression in *Escherichia coli* [14].

In this study, we hypothesized that *Salmonella typhimurium* flagellin (fliC) could be a stable fusion partner for HA1-2 and that the resulting fusion protein would be efficiently manufactured using an *E. coli* prokaryotic expression system and induce efficient immune response. We constructed a fusion protein by fusing HA1-2 with the N-terminus of fliC by physical linkers and then tested the resulting protein for TLR5-specific activity and immunoreactivity. Last, we demonstrated that the mice immunized intraperitoneally with the HA1-2-fliC fusion protein developed significantly higher HA1-2-specific serum IgG and hemagglutination inhibition (HAI) titers compared with the mice injected with HA1-2 alone.

## Methods

### Construction of recombinant plasmids

Viral RNA was extracted from the inactivated avian influenza A/chicken/Jiangsu/CZT4/2013 (H7N9) virus (Animal Infectious Disease Laboratory, School of Veterinary Medicine, Yangzhou University, Yangzhou, China) using TRIzol reagent (Invitrogen, USA), and viral cDNA was synthesized using Oligo dT primer (Takara, Dalian, China) according to the manufacturer’s instructions. Based on the HA sequence of influenza A virus (A/Hangzhou/1/2013(H7N9)) [GenBank: KC853766.1] and the sequence of *S. typhimurium* flagellin (fliC) [GenBank: CP001363.1], three pairs of primers were designed for amplifying the objective fragments (Table 1). The HA1-2 gene fragment was amplified from cDNA by PCR with forward primer HA1-2-F1 and reverse primer HA1-2-R1. Similarly, the HA1-2 fragment of the fusion gene was amplified using primers HA1-2-F2 and HA1-2-R2, and the full-length *fliC* gene was amplified using primers *fliC*-F and *fliC*-R from the template plasmid pET30a-*fliC*-WT [21]. Then, the HA1-2 gene was fused directly to the N-terminus of the *fliC* by overlap-PCR from the PCR products (HA1-2 and *fliC* gene) with forward primer HA1-2-F2 and reverse primer *fliC*-R. Both HA1-2 and HA1-2-*fliC* PCR products were cloned into the pCold vectors (Takara, Dalian, China), after being digested by *Sac*I/*Hin*dIII or *Eco*RI/*Xba*I in advance, generating the constructs pCold-HA1-2 and pCold-HA1-2-*fliC*, respectively. The resulting constructs were confirmed via DNA sequencing by Genscript (Nanjing, China).

**Table 1:** Polymerase chain reaction primers used in this study

Primers	Sequence	Restricted site
HA1-2-F1	5′-CCC*GAGCTC*AAAGGGAAAAGGACAGTTGACC-3′	*Sac*I
HA1-2-R1	5′-CCC*AAGCTT*GGCATCAACCTGTACT-3′	*Hin*d III
HA1-2-F2	5′-CCG*GAATTC*AAAGGGAAAAGGACAGTTGACC-3′	*Eco*RI
HA1-2-R2	5′-*CACCTCCGCTTCCACCTCCACC*GGCATCAACCTGTACT-3′	(Gly_4_Ser)_3_
*fliC*-F	5′-*AGGTGGAAGCGGAGGTGGTGGAAGC*ATGGCACAAGT CATTAATA-3′	(Gly_4_Ser)_3_
*fliC*-R	5′-CCG*TCTAGA*TTAACGCAGTAAAGAGAGGACG-3′	*Xba*I

### Expression and characterization of recombinant proteins

The recombinant proteins HA1-2 and HA1-2-fliC with a His-tag at the end of each of their N-terminuses were expressed in *E. coli* using a Cold Shock Expression System with pCold DNA (Takara, Dalian, China) according to the manufacturer’s instructions. Cell lysates were evaluated by SDS-PAGE. Soluble HA1-2-fliC was purified with a His•Bind Purification Kit (Novagen, USA), according to the manufacturer’s instructions. The purification of HA1-2 inclusion bodies was also performed with a His•Bind Purification Kit under the condition of 6 M urea, followed by dialysis in PBS. The purified proteins were resolved via SDS-PAGE, and Western blotting was performed using mouse polyclonal antibody specific for fliC (anti-fliC) or inactivated avian influenza A (H7N9) virus (anti-H7N9 virus).

### TLR5 activity of the fusion protein

TLR5 activity of the HA1-2-fliC fusion protein was evaluated by measuring the induction of IL-8 production by HEK 293-mTLR5 cells (Invivogen, USA). Cells were cultured in 96-well microtiter plates at a seeding density of 5 × 10^4^ cells in Dulbecco’s Modified Eagle’s Medium (DMEM) supplemented with 10 % fetal calf serum (FCS) and antibiotics. The next day, cells were treated for 5 h with 100 ng/ml of the fusion and control proteins. At the completion of the assay, supernatants were harvested, and IL-8 expression was evaluated by an enzyme-linked immunosorbent assay (ELISA), using Human IL-8 Ready-SET-Go!® ELISA Set (eBioscience, USA). Endotoxin was removed from the proteins HA1-2 and HA1-2-fliC by using the ProteoSpin™ Endotoxin Removal Kit Maxi for protein & peptides (Norgen, Canada) according to the manufacturer’s instructions, and the residual endotoxin level was measured using a chromogenic endpoint tachypleus amebocyte lysate (CE TAL) assay kit (Chinese Horseshoe Crab Reagent Manufactory Co., Ltd. China) according to the manufacturer’s instructions.

### Vaccination and blood sample collection schedule

**Fig. 1:**
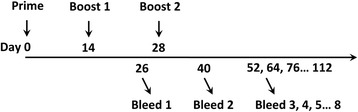
Vaccination and blood sample collection schedule. Groups of C3H/HeJ mice (*n* = 6) were vaccinated i.p. with three doses of HA1-2, HA1-2-fliC, or PBS on days 0, 14, and 28. The animals were bled on days 26 and 40, following the second and third immunization. In total, the animals were bled for about 3 months (84 days) at 12-day intervals, and the resulting serum samples were used to determine the serum antibody responses over time

Six-week-old female C3H/HeJ mice were purchased from the Biomedical Research Institute of Nanjing University, Nanjing, China. All animals were housed in isolators and fed a pathogen-free diet and water. The procedures described in this study were approved by the Committee on the Ethics of Animal Experiments of Yangzhou University, Yangzhou, China. Groups of C3H/HeJ mice (*n* = 6) were vaccinated intraperitoneally (i.p.) with doses of 15 μg HA1-2, 50 μg HA1-2-fliC (containing 15 μg HA1-2, according to the molecular weight), or PBS on days 0, 14, and 28. The animals were bled 12 days following the second and third immunizations (on days 26 and 40). Serum samples were subjected to hemagglutination inhibition assays and assays to determine the titers of HA1-2-specific IgG and its subtypes, IgG1 and IgG2a. Over the course of this schedule, the animals were bled for about 3 months at 12-day intervals, and serum samples were used to determine the time course of HA1-2-specific serum IgG responses (Fig. 1).

### ELISA

Serum titers of antigen-specific IgG, IgG1and IgG2a were determined by an indirect ELISA, as described previously [22]. Briefly, 96-well plates were coated with 1.5 μg/ml GST-tagged HA1-2 antigen overnight at 4 °C. After washing and blocking, serial dilutions of antiserum were added in triplicate and incubated for 2 h at 37 °C. Horseradish peroxidase (HRP)-conjugated goat anti-mouse IgG, IgG1, or IgG2a (Invitrogen, USA) were incubated for 1 h at 37 °C as the secondary antibody. 3, 3′, 5, 5′-tetramethybenzidine was used as a substrate to estimate the enzymatic activity. The reaction was stopped with 2 M H_2_SO_4_, and the absorbance was measured at 450 nm using a Microplate Reader (Bio-Tek EL 680, USA).

### HAI assay

All HAI assays were performed in V-bottomed 96-well plates. Serum samples collected from mice were treated with receptor destroying enzyme (RDE) II overnight, heat-inactivated (56 °C, 30 min), diluted in 96-well V-bottomed microtiter plates, and incubated with 4 HA units (HAU) of inactivated avian influenza A (H7N9) virus for 30 min at room temperature. After that, 1 % chicken erythrocytes were added, mixed briefly, and incubated for 30 min at room temperature. The highest dilution of serum that inhibited hemagglutination was considered the HAI titer.

### Statistical analysis

All results are expressed as the mean ± SEM unless otherwise stated. Serum titers of HA1-2-specific IgG, IgG1, and IgG2a were analyzed using log10 transformed data, and HAI serum titers were analyzed using log2 transformed data. The statistical significance between two groups was analyzed using an unpaired Student’s *t*-test with GraphPad Software 5.0 (San Diego, CA). *p* < 0.05 was considered statistically significant.

## Results

### Expression and characterization of the recombinant proteins

**Fig. 2:**
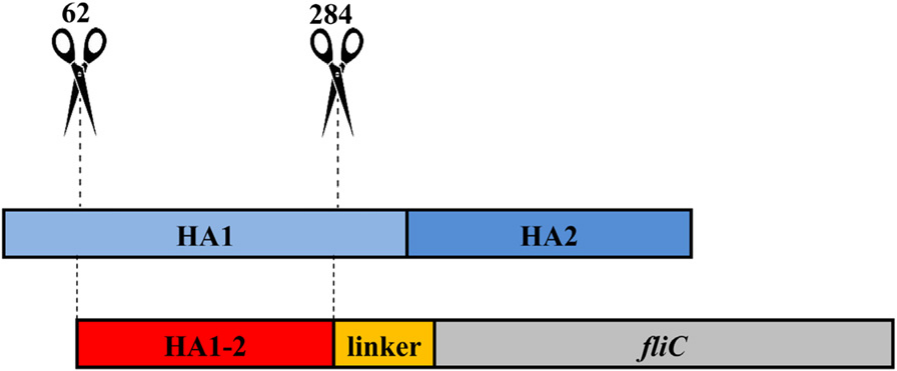
Schematic for the construction of HA1-2-*fliC*. The cDNA sequence encoding residues 62–284 of avian influenza A (H7N9) virus HA1-2 was cloned for expression in *E. coli*. Meanwhile, the HA1-2 gene was further fused to the N-terminus of the *fliC* gene to form HA1-2-*fliC* by a linker of flexible peptide (Gly_4_Ser)_3_

**Fig. 3:**
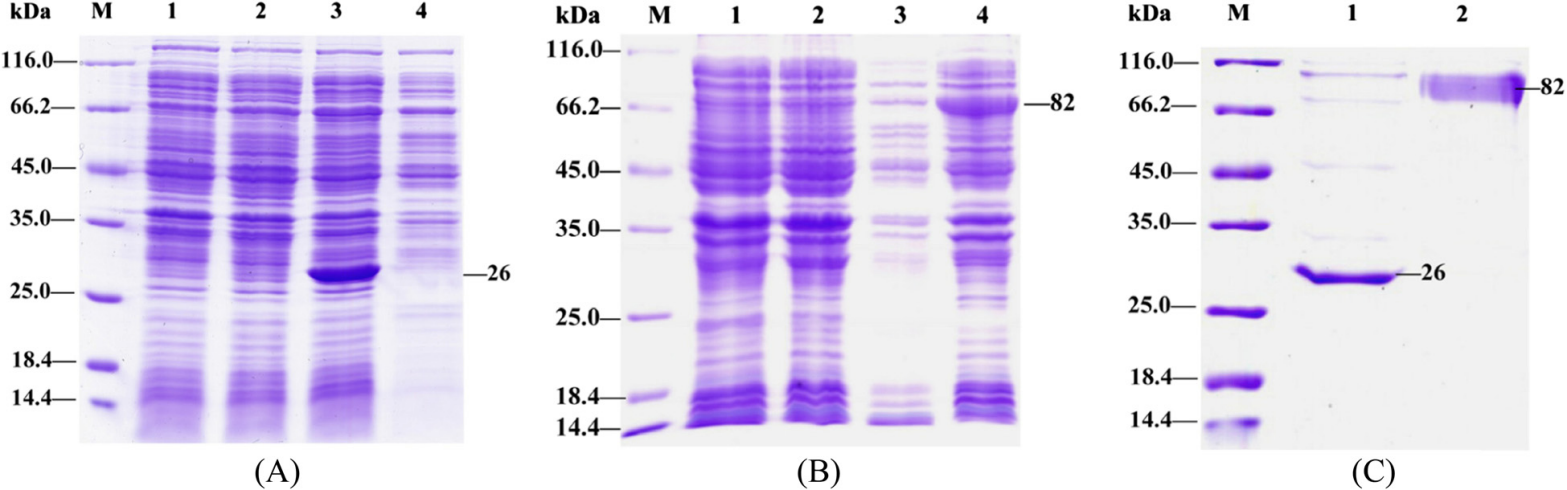
Protein expression and purification. **a** Expression of HA1-2. Lanes: M, protein marker; 1, product of BL21(DE3)(pCold) induced by IPTG; 2, product of BL21(DE3)(pCold-HA1-2) not induced; 3, lysate supernatant of BL21(DE3)(pCold-HA1-2) induced by IPTG; 4, inclusion bodies of BL21(DE3)(pCold-HA1-2) induced by IPTG. **b** Expression of HA1-2-fliC. Lanes: M, protein marker; 1, product of BL21(DE3)(pCold) induced by IPTG; 2, product of BL21(DE3)(pCold-HA1-2-*fliC*) not induced; 3, lysate supernatant of BL21(DE3)(pCold-HA1-2-*fliC*) induced by IPTG; 4, inclusion bodies of BL21(DE3)(pCold-HA1-2-*fliC*) induced by IPTG. **c** Protein purification. Lanes: M, protein marker; 1, HA1-2; 2, HA1-2-fliC

**Fig. 4:**
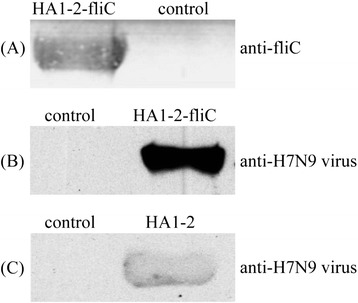
Western blotting analyses. **a** HA1-2-fliC fusion protein was detected using an anti-fliC polyclonal antibody. **b** HA1-2-fliC fusion protein was detected using an anti-influenza A (H7N9) virus (anti-H7N9 virus) polyclonal antibody. **c** HA1-2 protein was detected using an anti-influenza A (H7N9) virus (anti-H7N9 virus) polyclonal antibody

To create a fusion protein of HA1-2 and fliC, a fragment containing the HA1-2 gene fused to the N-terminus of *fliC* gene was designed (Fig. 2). The HA1-2-*fliC* and HA1-2 genes were cloned into pCold vectors to add a His-tag to each target protein at the N-terminus. The resulting proteins were successfully expressed in *E. coli* BL21, strain DE3 (Fig. 3a and b). Subsequent purification of HA1-2 and HA1-2-fliC by His-tag affinity chromatography produced proteins with the expected molecular weights of 26 kDa and 82 kDa, respectively (Fig. 3c). The immunoreactivity of the purified HA1-2-fliC was confirmed by Western blotting using anti-fliC or anti-H7N9 virus polyclonal antibodies (Fig. 4a and b). Similarly, purified HA1-2 was also analyzed by Western blotting using an anti-H7N9 virus polyclonal antibody (Fig. 4c).

### TLR5 activity of the fusion protein

**Fig. 5:**
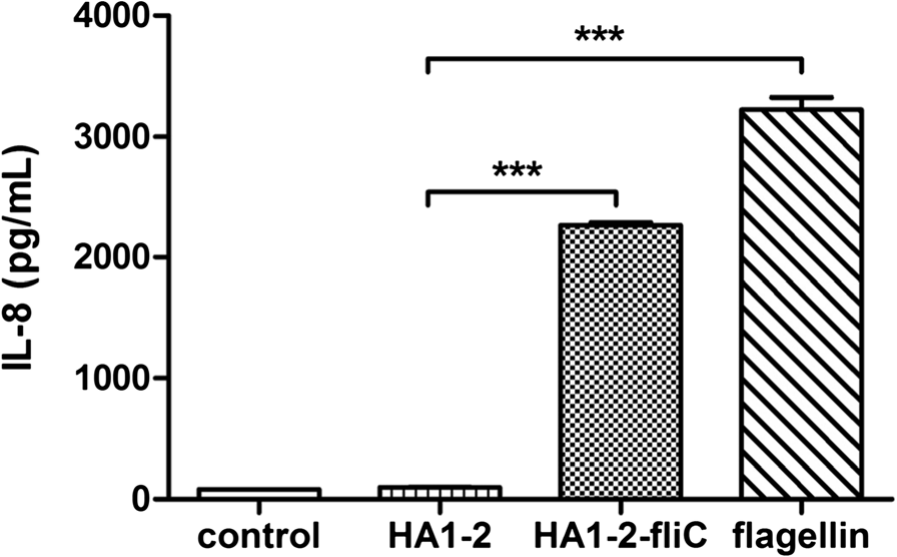
TLR5 activity of the fusion protein. Each of the proteins was tested for TLR5 activity using HEK-293-mTLR5 cells. HA1-2-fliC was compared to HA1-2 alone, using 100 ng/ml of each, and flagellin (Enzo Life Sciences, USA) was used as a positive control. Data are presented as mean ± SEM of IL-8 (pg/ml); *p < 0.05, **p < 0.01, *** *p* < 0.001

The ability of the fusion protein to activate TLR5 was assessed using an *in vitro* assay. HA1-2-fliC induced a significantly stronger IL-8 secretion (about 2,265 pg/ml) than the HA1-2 control (99 pg/ml) (*p* < 0.001), and a slightly lower IL-8 level than the positive control (about 3,226 pg/ml) (Fig. 5). To exclude the possibility that our results were due to contamination by endotoxin in the IL-8 assay, the endotoxin level was tested, and it was determined to be less than 0.01 EU/μg.

### Immunogenicity of the recombinant proteins

**Fig. 6:**
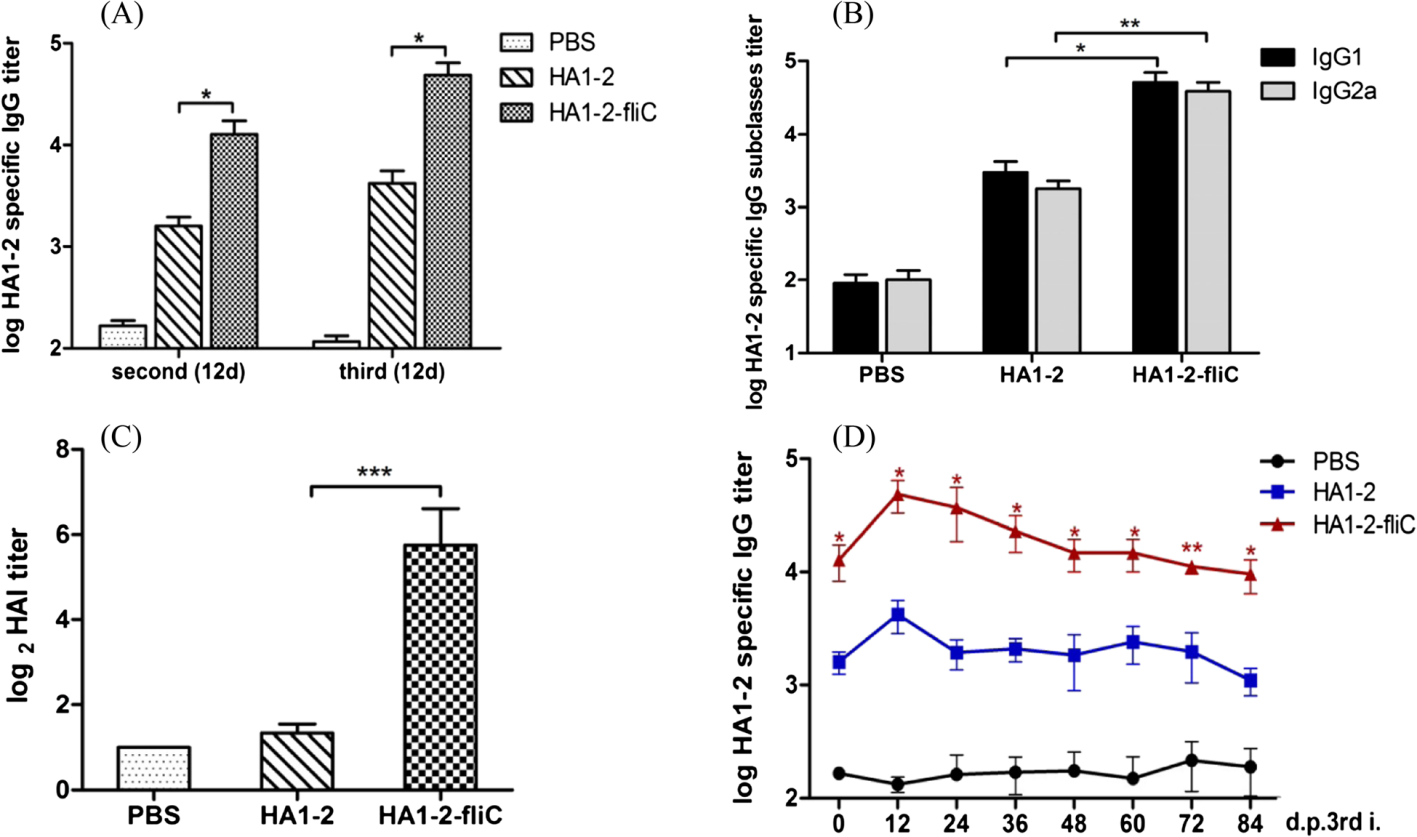
Immunogenicity of fusion protein. Groups of C3H/HeJ mice (*n* = 6) were vaccinated i.p. with three doses of HA1-2, HA1-2-fliC, or PBS on days 0, 14, and 28. The animals were bled 12 days following the second and third immunization. Antibody titers were measured by ELISA or HAI assay. **a** HA1-2-specific IgG titers. Serum samples of each group were collected at 12 days post-second and -third immunization (on days 26 and 40). **b** HA1-2-specific IgG subtype (IgG1, IgG2a) titers. Serum samples of each group were collected at 12 days post-third immunization (on day 40). **c** HAI titers. Serum samples of each group were collected at 12 days post-third immunization (on day 40). **d** The longevity of HA1-2-specific IgG. Serum samples of each group were collected for 3 months at 12-day intervals after the third immunization (days post-third immunization, d.p.3rd.i). All Data are presented as mean ± SEM; *p < 0.05, **p < 0.01, *** *p* < 0.001

Humoral immune responses induced by i.p. immunization with the purified proteins were assessed by measuring the IgG titers in the serum of vaccinated mice via an indirect ELISA. Each group of C3H/HeJ mice was bled on days 26 and 40. HA1-2-fliC induced significantly stronger (*p* < 0.05) HA1-2-specific IgG responses than HA1-2 or PBS, particularly on day 40 (12 days after the third immunization). The HA1-2-specific IgG titers induced by HA1-2-fliC after the third immunization were about 4-fold higher than those following the second immunization (Fig. 6a). Additionally, immunization with HA1-2-fliC induced significantly higher IgG1 (*p* < 0.05) and IgG2a (*p* < 0.01) titers than immunization with HA1-2 or PBS. The IgG1 and IgG2a average titers of HA1-2-fliC vaccinated mice were 5.1 × 10^4^ and 3.8 × 10^4^, respectively, and these amounts are not significantly different from one another (Fig. 6b). These data indicate that vaccination with HA1-2-fliC induced a relatively balanced IgG1 and IgG2a response.

A standard HAI assay was conducted to test the ability of HA1-2-fliC to generate HAI antibodies. HAI antibody titers were measured at 12 days after the third vaccination for each group. The HA1-2-fliC group had significantly higher HAI antibody titers than the HA1-2 (*p* < 0.001) or PBS groups (Fig. 6c).

### The longevity of HA1-2-specific serum IgG

To monitor the longevity of HA1-2-specific IgG antibodies induced by the HA1-2-fliC protein in mice, the animals were bled for about 3 months (84 days) at 12-day intervals after the third immunization, and ELISAs were performed on the serum samples to determine the serum titers. We analyzed the time course of HA1-2-specific antibodies induced by the HA1-2 and HA1-2-fliC proteins for 84 days (Fig. 6d). The IgG titers in the HA1-2-fliC group reached the highest levels on day 12 post-immunization, and then fell slightly over the next 3 months. HA1-2-fliC induced approximately 5–18 fold higher IgG titers than HA1-2 over the different time points. Importantly, the HA1-2-specific IgG titers remained high, even on day 84, suggesting that HA1-2-fliC vaccination elicited robust HA1-2-specific serum IgG titers that lasted for at least 3 months in mice.

## Discussion

Expression of proteins in a prokaryotic system such as *E. coli* could be substantially less expensive and allow for a more rapid production of vaccines compared with conventional methods [23]. Previously, improvements in influenza vaccine production by the industry have focused on cell culture. The cell culture-based approach alleviates the significant manufacturing issues associated with egg-based manufacturing, but it does not improve production efficiency [14]. Recent data show that the glycosylation pattern of HA does not impact the antibody response, suggesting that glycosylation is not required for appropriate folding of the molecule [24]. That finding is supported by our study, which demonstrates that the HA1-2-fliC protein can be successfully and efficiently expressed using an *E. coli* prokaryotic system (Fig. 3), and it elicits a robust antibody response (Fig. 6).

Early vaccine formats fused the HA globular head to the C-terminus of flagellin, replaced domain 3 of flagellin, or placed one copy of the HA head at the C-terminus while using a second copy to replace domain 3 [25]. Importantly, the vaccine format not only affects the immunogenicity of the vaccine antigen, it also affects the safety of the vaccine in preclinical models [26]. Delaney et al. have found that replacement of the hypervariable region of flagellin with the vaccinia virus antigen L1R results in a vaccine that does not generate antibody against native L1R. However, if L1R is fused at the N-terminus of flagellin, antibody can be generated against native L1R [27]. Therefore, in this study, we extended the flagellin-based vaccine format by fusing the HA1-2 antigen to the N-terminus of flagellin. SDS-PAGE analysis confirmed that the fusion protein HA1-2-fliC was efficiently expressed in an *E. coli* expression system (Fig. 3a and b). Additionally, the immunoreactivity of HA1-2-fliC was confirmed via Western blotting using anti-fliC or anti-H7N9 virus polyclonal antibodies (Fig. 4a and b), suggesting that the HA1-2 and fliC moieties of the fusion protein are faithfully refolded to take on the native conformations of the parent two proteins.

Based on the literature, interaction of fliC with TLR5 leads to the secretion of pro-inflammatory cytokines, such as IL-8, in epithelial cells [28]. In our study, TLR5-dependent IL-8 secretion was induced by the HA1-2-fliC fusion protein, suggesting that it stimulates potent TLR5 signaling (Fig. 5). This finding is similar to that from a recent study showing that the fusion protein flagellin β-defensin-3 (FBD3) was also recognized by TLR5 expressed on target cells and stimulated the secretion of IL-8 [29]. These results suggest that the protein made from the C-terminal HA1-2 fused with the N-terminal fliC retains its TLR5 binding capacity.

Researchers have previously demonstrated the excellent immunogenicity and efficacy of the vaccine platform (the physical linkage of vaccine antigens to flagellin) when applied to either seasonal H1N1 or highly pathogenic avian influenza H5N1 in animal models [14, 15]. In these studies, the fusion proteins elicited remarkably high antigen-specific IgG and protective HAI titers, which protected mice against disease and death in a lethal challenge model. In our study, HA1-2-fliC also induced significant and robust HA1-2-specific IgG responses, particularly on day 12 after the final immunization, with a mean of 4.8 × 10^4^ (Fig. 6a). Thus, presentation of HA1-2 in the context of a flagellin fusion protein significantly enhances the potency of HA1-2 as an immunogen. Our results are consistent with the findings that flagellin-L1R and flagellin-B5R fusion proteins are effective in eliciting immune responses against vaccinia virus [27] and that flagellin-containing VLPs (containing A/PR8/34(H1N1) HA, matrix protein (M1)) elicited higher specific IgG responses than standard HA and M1 VLPs [30].

Some studies [31, 32] reported that co-delivery of flagellin with an antigen resulted in Th2 responses *in vivo*. However, another study suggested that co-immunization of flagellin with inactivated foot-and-mouth disease virus (FMDV) antigen induced Th1 polarization of the immune system [33]. Several other reports of antigen-flagellin fusion proteins showed both Th1 and Th2 type immune activation by flagellin in a BALB/c mouse model [34]. Dziadek et al. found that the levels of Th1-type cytokines and lymphoproliferation were dependent on the vaccine composition and the genetic background of the mice [35]. Sack et al. also found BALB/c F9(−/Y) mice had a Th2 skewed response and C3H/HeJ F9(−/Y) mice had a mixed Th1 and Th2 response [36]. Moreover, the cytokine profile in C3H/HeJ females was a mixture of Th1 and Th2 whilst a mainly Th1 profile was observed in C57BL/6 mice during the allogeneic and syngeneic vaccination against prostate cancer [37]. In our study, both HA1-2-specific IgG1 and IgG2a titers in the HA1-2-fliC group were significantly higher than those in the HA1-2 group (Fig. 6b), suggesting that HA1-2-fliC elicits a higher and more balanced Th1 and Th2 response than HA1-2 alone in a C3H/HeJ mouse model. Consistent with our study, C3H/HeJ mice injected with the multimeric VLP vaccine showed a strong and long-lasting immune response against a C-terminal fused epitope with a balanced Th1/Th2 response [38].

Vaccination that induces long-term immunity is still regarded as the best means of protection against influenza. An essential requirement of any vaccine is the induction of long-term protective immunity [39]. In a previous study, blood samples of adults were collected on days 0, 7, and 30 after immunization with trivalent inactivated influenza vaccine (TIV) or live-attenuated influenza vaccine (LAIV) vaccines. In these subjects, the effector IgA and IgG antibody secreting cell (ASC) responses appeared in the circulation with a sharp peak around day 7, and quickly disappeared thereafter (on day 30) [40]. Researchers have also evaluated the effector response in adults and 5- to 9-year-old children after TIV immunization (days 27 to 47) and found that very few subjects had influenza virus-specific IgG ASC [41]. In our research, the IgG titers induced by HA1-2-fliC reached the highest levels on day 12 post-final boost, and the HA1-2-specific IgG titers in these mice remained high, even on day 84 (Fig. 6d). Our results are similar to those from an H5N1 influenza virus study in which flagellin-HA vaccines also elicited robust HAI antibody responses for 3 months [25].

HAI antibody activity remains a World Health Organization accepted correlate of protection against influenza [42]. Song et al. found that the STF2R0.HA1-2 (created by using H5N1 HA1-2 to replace the domain D0 of *S. typhimurium* fljB, STF2) construct failed to elicit significant levels of serum HAI antibodies following either two or three immunizations, while STF2R3.HA1-2 (created by using H5N1 HA1-2 to replace the domain D3 of STF2) elicited the highest HAI titers with geometric mean titers of 63 and 35 following three and two immunizations, respectively [15]. Here, the significant HAI titers (average 54, following three immunizations) that were elicited by HA1-2-fliC confirmed that it has a higher immunogenicity than HA1-2 alone (Fig. 6c). Based on these results, we cautiously predict that the HA1-2-fliC subunit vaccine could provide effective protection against H7N9 influenza virus infection.

Recently, it was reported that a *Vibrio vulnificus* flagellin (FlaB) served as a mucosal adjuvant to enhance tetanus toxoid-specific antibody responses in both systemic and mucosal compartments [43]. Additionally, HIV antigen p24 replacement of domains D2 and D3 in non-pathogenic *E. coli*-derived flagellin (KF) promoted mucosal IgA production, creating a potentially promising mucosal adjuvant [44]. Based on these intriguing findings, our future work will explore the mucosal adjuvant activity of *S. typhimurium* flagellin for influenza H7N9 vaccine development.

## Conclusions

Our study has shown that a HA1-2-fliC recombinant subunit vaccine was efficiently expressed in an *E. coli* prokaryotic system, and it induced a robust antigen-specific antibody response that was maintained at maximal levels in the serum for at least 3 months. This vaccine also induced a functional antibody response (HAI). In summary, the fliC fragment of the fusion protein successfully acts as an adjuvant, and improves the immunogenicity of the recombinant subunit vaccine, making this a viable strategy for creating successful H7N9 influenza vaccines.
